# Weight Loss and Fat Metabolism during Multi-Day High-Altitude Sojourns: A Hypothesis Based on Adipocyte Signaling

**DOI:** 10.3390/life12040545

**Published:** 2022-04-06

**Authors:** Stephan Pramsohler, Martin Burtscher, Linda Rausch, Nikolaus C. Netzer

**Affiliations:** 1 Hermann Buhl Institute for Hypoxia and Sleep Medicine Research, 83043 Bad Aibling, Germany; nikolaus.netzer@eurac.edu; 2 Institute of Sports Science, University Innsbruck, 6020 Innsbruck, Austria; martin.burtscher@uibk.ac.at (M.B.); l.rausch@uibk.ac.at (L.R.); 3 Department Medicine, Division of Sports Medicine and Rehabilitation, University Hospitals, 89070 Ulm, Germany; 4 Institute of Mountain Emergency Medicine, Eurac Research, 39100 Bozen, Italy

**Keywords:** high altitude, mountaineering, fatty diet, nutrition, fat metabolism

## Abstract

Several publications and random observations have reported weight loss in high-altitude sojourners of both sexes. This could be a result of multiple adaptations, which hypoxia and mountaineering provoke on a cellular and organic level. Several publications have discussed the effect on appetite-regulating hormones to be one of the main contributing factors. We aimed to review the available data and show the current state of knowledge regarding nutritional aspects in high altitude with a special focus on fatty dietary forms. To reach this aim we conducted a literature search via PubMed according to the PRISMA 2020 protocol to identify relevant studies. We found that very few studies cover this field with scientifically satisfying evidence. For final analysis, reviews as well as papers that were not clearly related to the topic were excluded. Six articles were included discussing hormonal influences and the impact of exercise on appetite regulation as well as genetic factors altering metabolic processes at altitude. Leptin expression seems to be the biggest contributor to appetite reduction at altitude with an initial increase followed by a decrease in the course of time at high altitude. Its expression is greatly dependent on the amount of white adipose tissue. Since the expression of leptin is associated with an increased β-oxidation of fatty acids, a high-fat diet could be advantageous at a certain time point in the course of high-altitude sojourns.

## 1. Introduction

Weight loss at high altitude is commonly observed and becomes especially pronounced with increasing altitude and duration of exposure [1]. Appropriate energy intake is considered as the major determinant of body weight loss in hypoxia, i.e., at high altitude. When exercising at high altitude, energy requirements increase and meeting the needs of energy intake becomes particularly challenging (Westerterp and Kayser 2006). When climbers ascend to high and especially to extreme altitudes, they are usually not able to adjust to the needs of energy requirements, inevitably resulting in body weight loss, partitioned between fat and lean mass [2]. Westerterp-Plantenga and colleagues performed a seminal study in order to identify the extent and reason of reduced energy intake on weight loss during a simulated (hypobaric hypoxia) ascent to the top of Mount Everest (8848 m a.s.l.) [3]. Although palatable food was provided ad libitum, and stresses like cold exposure and exercise were avoided, an average body mass loss of 5 kg was observed, which was accompanied by reduced energy amounting to 4.2 MJ/day. Up to about 6000 m a.s.l., study participants did not suffer from acute mountain sickness and the reduction in energy intake (meal size reduction) was a consequence of a more rapid occurrence in satiety. At simulated altitudes above 7000 m a.s.l. however, when acute mountain sickness developed, the authors identified uncoupling between hunger and desire to eat as the primary reason for reduced energy intake and disturbance of energy balance requirements [3]. Mechanisms of diminished appetite at high altitude, not related to acute mountain sickness, may include the elevation of leptin, a key hormone involved in the control of satiety and energy balance [4]. In addition, and especially in individuals suffering from acute mountain sickness, cytokines like interleukin-6 and tumor necrosis factor-α may contribute to anorexia [5]. Notably, studies performed at real altitude may be confounded by other environmental factors than hypoxia, e.g., extremely low temperatures with wind-chill, psychological stress and reduced variety of available foods. Energy loss associated with those conditions, but also changes in the basal metabolic rate due to cold exposure, may considerably affect energy expenditure, as the basal metabolic rate accounts for about two thirds of the daily energy expenditure. These effects have been demonstrated in an early study by Nair and colleagues (1971) [6]. In times that are more recent, hypoxia effects on energy balance have been studied under standardized conditions in normobaric hypoxia, e.g., hypoxia chambers or tents. It was demonstrated that even a 10-day exposure of healthy individuals to normobaric hypoxia without exercise resulted in negative energy balance, which was primarily due to diminished energy intake [7]. Moreover, intermittent training in normobaric hypoxia was suggested to result in more weight loss of young obese adults than the same training in normoxia [8]. Hypoxia not only affects appetite, but seems also to cause a shift in substrate use favoring increased glucose oxidation [9]. This might be advantageous due to the increased metabolic economy (higher yield of adenosine triphosphate (ATP) per mole of oxygen) resulting from glucose compared to fat oxidation, which was supported by the observation of lower oxygen uptake at the same workload after acclimatization to hypoxia/high altitude [9]. However, the oxygen-saving benefit of carbohydrates is likely outweighed by limited carbohydrate stores [10]. Beside the energy imbalance during prolonged high altitude exposures, the loss of body water contributes to weight loss [11]. Obviously, effects of high altitude on energy balance and related weight loss are extremely complex, even when considering solely the effects resulting from hypoxia.

Several studies have dealt with changes of body weight during prolonged sojourns to high altitudes [12,13,14,15]. Even though the fitness industry has already started to promote hypoxic training in several variations as a useful weight loss strategy, scientific publications do not support a definitive statement. The occurrence of weight loss at altitude seems to be driven by multiple factors such as nutrition, increased energy expenditure, hormone based changes in appetite and genetic factors in addition to the hypoxic stimulus [12]. The use of the single factor hypoxia as standalone prevention or rehabilitation strategy excludes the other potentially rather important contributors to weight loss at altitude [12]. The reduction of appetite in combination with a higher energy expenditure seems to be an especially crucial factor. Acute short term exposure to altitude seems to upregulate leptin levels via the hypoxia inducible factor HIF 1α and contributes to reduced appetite [13,16]. This seems to be greatly dependent on the duration of hypoxic exposure and accounts for a greater weight loss in the first weeks of altitude sojourns [17,18]. The time, duration and dose factors of this mechanism and its implications for an adapted nutrition for altitude sojourns in the human metabolism are not completely understood [19]. Research at a cellular level on isolated human adipocytes in their reaction to hypoxia most often concentrates on HIF-mediated inflammatory signaling in a long-term hypoxic state in regard to obesity, cancer or cardiovascular diseases [20,21,22,23]. However, there is some research on isolated human adipocytes, which could give hints for a time dose–response-curve of leptin and GLUT1 up- and downregulation mediated by levels of HIF 1 α in hypoxia and its effects on appetite of high fat saturated nutrients at high altitude [24]. A study on isolated human adipocytes by Wang, Wood and Trayhurn primarily investigated dysregulation of inflammatory adipokines by hypoxia. However, their findings on the time curve of leptin upregulation and downregulation in relation to the level of HIF 1α also give some insight on the effect of hypoxia for reduced and increased appetite for fat and the resulting weight loss as well as the responding weight regain at high altitude.

Any knowledge of the implications on cellular hypoxia reactions of nutrient metabolism in humans at high altitude could allow the maintenance of a higher physical and mental performance during stays at altitude including professional operations and recreational activities, and add to our understanding of appetite regulation in general. Especially during prolonged exposures, an imbalance of energy expenditure and energy intake causes a depletion of body fat stores. This misbalance seems to increase with increasing altitude [19]. De Glieszinski et al. investigated the effect of prolonged hypoxia on adipose tissue lipolysis during a stay in a hypobaric altitude laboratory. They found that after an initial depletion of fat stores, after prolonged hypoxia there was a tendency to preserve fat stores by a downregulation of beta3 adrenergic receptors to chronic adrenergic activation [25]. Furthermore, there is some evidence for malabsorption of carbohydrates exceeding altitudes of 5500 m a.s.l., which is hypothesized to account for weight loss even though energy intake is adapted to the corresponding altitude [26]. This could likely indicate that a high saturated fat diet might be advantageous during high-altitude sojourns with the perspective of minimizing weight loss and maximizing energy uptake. Studies covering the area of appetite regulation and weight loss at altitude are scarce and do not give a conclusive picture of how different forms of diet affect metabolism and performance at altitude. Therefore, we aimed to review the available data and show the current state of knowledge regarding nutritional aspects in high altitude with a special focus on fatty dietary forms.

## 2. Materials and Methods

**Figure 1:**
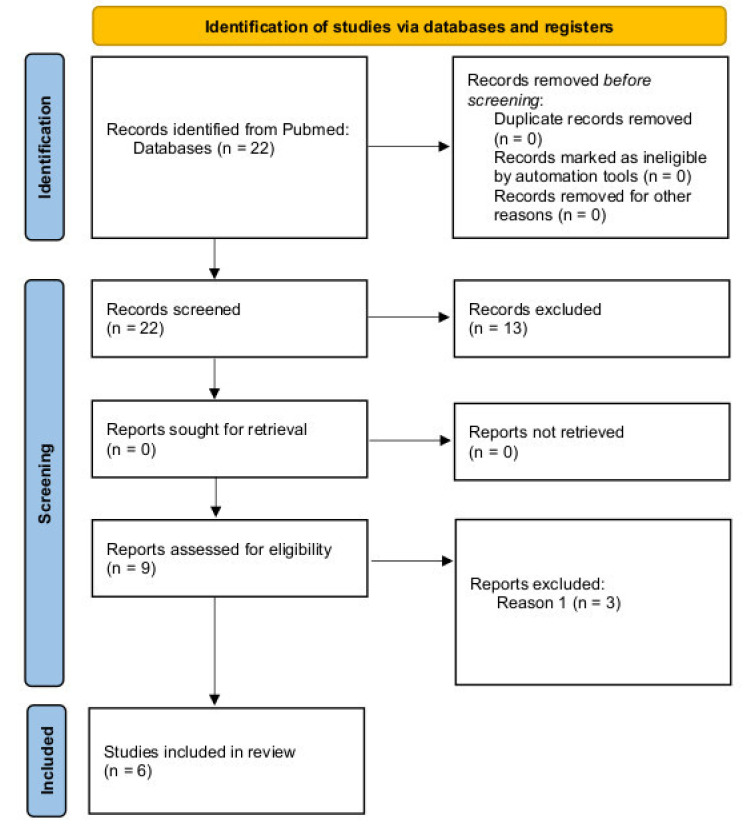
Flow diagram according to PRISMA 2020 guidelines.

A literature search was conducted according to preferred reporting items for review and meta-analysis protocols (PRISMA-2020) statement. Via PubMed searches, relevant literature for this review was identified. The keywords for the search were “altitude” as major topic and “fatty diet” and “nutrition”. Review articles were excluded. There was no restriction regarding study design. After this search, a total of 22 papers were identified. The same reviewer reviewed all studies. After title and abstract evaluation, 13 studies were excluded due to lack of coherence with the topic or because they were identified as reviews. After assessing full-text articles for eligibility, another three papers were identified as reviews or as off topic. Finally, six articles were eligible for inclusion in this review and selected for analysis. Figure 1 shows a flow diagram according to PRISMA 2020 protocols displaying the process of literature identification, screening, eligibility and inclusion.

After the final evaluation of the six included articles (Table 1), four categories were identified as associated with nutritional aspects at altitude and fat metabolism. One study investigates the impact of ghrelin on altitude-related appetite reduction. One study shows the impact of exercise on altitude-induced appetite changes. One study occupies the field of changed metabolism and fuel utilization at altitude. Three studies investigate genetic factors for altitude resistance in Andean and Tibetan populations and show the impact of prolonged high-altitude sojourns on obesity.

## 3. Results

After the study selection process via the PRISMA 2020 protocol, four relevant categories of study were identified. Matu et al. investigated hormonal changes during a high-altitude trek in 12 participants and its influence on appetite perception and body composition. Even though the study is well done, unfortunately, in this study leptin levels were not measured (Section 3.1). Debevec et al. investigated the effect of exercise in addition to a 10 days normobaric hypoxia confinement at an equivalent of 4000 m a.s.l. A main factor limiting this study was the significant difference in food intake between the exercise and the control group. Therefore, the authors cannot be sure if the lack of significant differences between the two groups arise from a negative energy balance in the exercise group (Section 3.2). McClelland et al. investigated the effect of altitude on fuel utilization during exercise in rats. In contrast to previous studies, they found no additional effect of altitude exposure on exercise metabolism. The study group used a new approach and tried to exclude the influence of hypoxic environments on the maximum oxygen capacity (Section 3.3). Three studies investigated alterations in high altitude residents in metabolic parameters. Horsecraft et al. compared metabolic mechanisms between lowlanders and the Sherpa. Barbacini et al. investigated the phenomenon of a higher prevalence of metabolic syndrome in an Andean population and its link to metabolic markers. Voss et al. found a lower risk for the diagnosis of obesity in military personnel stationed above 1960 m. (Section 3.4).

### 3.1. Hormonal Appetite Reduction

Matu et al. investigated the impact of altitude ascent on the leptin antagonist ghrelin. Leptin and ghrelin are opponents in appetite regulation and their levels during high-altitude sojourns are thought to influence energy intake and fat metabolism [14,27,28]. HIF is a transcriptional factor that is unstable in the presence of oxygen. In hypoxic situations such as high-altitude sojourns or also in cancer patients, HIF activates a series of adaptive mechanisms leading to the commonly known effects of hypoxia [17,29]. The consequences of this activation result in an enhanced expression of erythropoietin and therefore an increase in red cell mass as well as in the expression of vascular endothelial growth factors (VEGFs). In addition, it seems to stimulate the expression of leptin and therefore influences appetite regulation [13]. Elevated leptin levels have been shown to occur especially in the first days of altitude exposure [24]. Since leptin is subject to feedback regulation, the time point of measurements may play a crucial role in future studies [24]. The different levels of leptin in the course of a day and the dependency of the overall expression capacity in relation to white adipose tissue may have accounted for the different study outcomes in the past [30]. Matu et al. found that next to the elevation of leptin, total ghrelin is also influenced with ascent to altitude and here especially the concentration of acylated ghrelin is diminished and seems to be the driving factor for lower total ghrelin levels. Matu et al. found a significant decrease in energy uptake during a high-altitude trek lasting 14 days to a maximum of 5300 m a.s.l. Even though also a significant reduction in appetite as well as a reduction in body fat could be observed, no significant correlation between appetite perceptions could be found in their study. This suggests that there are further factors influencing altitude-induced weight loss and changes in body composition. Enhanced leptin expression may play the more important role than depressed acylated ghrelin expression at altitude [30]. Nevertheless, enhanced leptin expression seems to reduce appetite especially in the first several days of altitude exposure, whilst it normalizes after some time of acclimatization [18,31].

Generally, in the first days of altitude exposure, leptin levels rise and result in reduced appetite, while the decrease of ghrelin levels does not seem to have as much influence.

### 3.2. Exercise-Induced Appetite Reduction

Exercise is known to modulate appetite [32]. Acute exercise results in less appetite and chronic exercise enhances appetite in the long run. This is probably due to a sympathetic activation during acute exercise resulting in a downregulation of all systems related to food uptake and digestion. On the other hand, chronic exercise enhances energy expenditure and metabolic rate at rest and provokes conclusively more appetite in the long run [33]. Therefore, exercise during altitude accent is thought to contribute to altitude-related decrease of appetite. Debevec et al. investigated the impact of these exercise-related appetite modulations on weight loss during 10 days of exposure to normobaric hypoxia equivalent to 4000 m a.s.l. One group conducted moderate exercise (60 min/day) representing an ascent and the other group was not allowed to exercise during the 10 days of confinement. The measurements of total ghrelin, peptide YY, and glucagon-like peptide-1, leptin, adiponectin, expired CO_2_, as well as perceived appetite ratings, showed no significant differences. Nonetheless, some changes could be observed such as decreased postprandial glucose response and reduced cholesterol levels with a shift towards healthier lipid profiles [32]. Since an exercise length of only 60 min per day does, in our opinion, not represent the demands of common high-altitude treks, the low exercise duration of one hour could be the cause for the missing effects on appetite-related parameters.

According to these findings, exercise does not seem to play a major role in appetite reduction during altitude sojourns.

### 3.3. Energy Expenditure and Related Fuel Utilization

It has been found that metabolism and fuel utilization in high altitude changes due to higher utilization of carbohydrate metabolism [34]. McClelland et al. postulate that this outcome is due to the altitude-induced reduction of the maximal aerobic capacity (VO2 max) and not so much on a general change in metabolism [10]. Therefore, they compared 32 female Wistar rats randomly assigned to two groups. One group stayed at sea level while the other group stayed at an equivalent of 4300 m a.s.l. simulated hypobaric altitude. After an acclimatization period of at least 10 weeks, the measurements of fuel utilization during exercise begun. Both groups completed their exercise at sea level with the same intensities. Due to the diminished oxygen availability, VO2 max is typically reduced in high altitude. Therefore, McClelland et al. tried to exclude this factor by conducting the measurements after acclimatization at sea level [10]. Previous studies used the same training parameters in both hypoxic and normoxic conditions, which leads to a higher training intensity in hypoxia [35]. This higher training intensity leads, as shown by McClelland et al., to a higher carbohydrate metabolism in both conditions. Therefore, it seems that fuel utilization in high altitude depends much more on work intensities than on the hypoxic environment itself [10].

Altitude metabolism seems not to be significantly different from metabolism at sea level with regard to the influence of training intensity.

### 3.4. Genetic Adaptions to Hypoxia and Long-Term Sojourns

**Figure 2:**
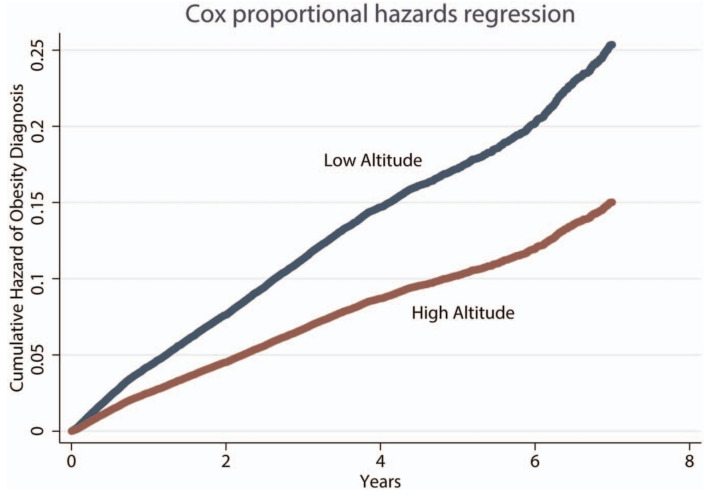
The displayed data show the cumulative hazard of obesity diagnosis (low vs. high altitude) in military personnel based on Cox proportional hazards model adjusted for enlistment BMI, sex, race, occupation, time in service, branch of service, housing allowance, and age. Those stationed at high altitude >1.96 km a.s.l. (red line) for a median of 3.2 years show a lower risk for obesity diagnosis in an observation period of seven years than those stationed at low altitudes (red line) [40].

It has been found that populations living for generations in high altitude not only show different adaptions than lowlanders but also than other permanent altitude residents [36,37]. Tibetan Sherpa are known to tolerate high altitude much better than lowlanders which is probably caused by a genetic selection over generations, favoring altitude-related adaptations [38]. Horscroft et al. compared the metabolic basis of Sherpa altitude adaptation during a gradual ascent to Mount Everest basecamp (5300 m a.s.l.) with 15 representatives of the Sherpa population compared to that of 10 lowlanders. They found that Sherpa also have adaptations regarding metabolism, such as improved muscle energetics as well as a better protection to oxidative stress. It seems as if Sherpa have a lower fatty acid β-oxygenation and a higher tolerance of lactate [38]. The combination of both suggests a higher use of carbohydrates as well as a conservation of fatty acids in the Sherpa population, which could enhance their resistance to weight loss as well as cold temperatures during high-altitude ascents. It has been shown that Andeans have developed different genetic adaptations for high-altitude tolerance [36]. Barbacini et al. describe a central role of sphingosine-1-phosphate as contributor to a high prevalence of metabolic syndrome in Andeans, although the metabolic mechanisms behind it are not yet completely understood [39]. The predisposition for obesity in Andeans, associated with an unfavorable high-density lipoprotein (HDL-C) and low-density lipoprotein (LDL-C) ratio, as well as high triglyceride levels, seems to be a standalone phenomenon in altitude. Most studies regarding altitude adaptations in various other population groups find a reduction in the occurrence of obesity and metabolic markers following high-altitude sojourns [12]. Voss et al. analyzed the medical records of overweight military members at risk for obesity, either stationed at high altitude (above 1960 m) or at sea level (below 980 m). They found a reduced prevalence of obesity diagnosis after high-altitude sojourns in a very large population (98,009 participants) with a median altitude exposure of 3.2 years. Results show that military service members stationed at altitude have a 41% lower hazard rate of obesity than the control group, as shown in Figure 2 [40].

There seem to be genetic factors altering metabolism at altitude. Especially in Tibetans, a lower β-oxygenation of fatty acids and a higher lactate tolerance improve metabolism for high-altitude sojourns.

## 4. Discussion

To our knowledge, this review is one of the first to summarize changes in appetite regulation and energy expenditure during high-altitude sojourns. The main contributing factors to weight loss at altitude seem to be a reduction in appetite due to a higher leptin secretion and a higher working intensity. Leptin downregulates appetite, especially during the first 14 days of high-altitude sojourns with a peak around the 10th day. As we have shown in a previous paper, the initial increase of leptin expression seems not to persist long term, probably correlating with a certain degree of altitude acclimatization [18,41]. This increase of leptin availability seems to be the driving factor for weight loss in the first days of high-altitude sojourns [42]. As outlined in a recent review, hypoxia importantly modulates leptin levels, in particular with prolonged hypoxia exposure, but several other hormonal markers may contribute [43]. Especially during acute hypoxia, the decrease of acylated ghrelin concentration may lead to reduced appetite [43].

Another rather important factor seems to be the hypoxia-induced increase in metabolic rate. Especially in the first days of acclimatization, physical parameters such as heart rate, breathing frequency and, therefore, work intensity are elevated even at rest [35,44]. Additionally, physical exercise, cold and shivering adds significantly to a higher energy expenditure [45]. This accounts for a greater energy expenditure and a metabolic shift towards an increased β-oxidation [32,46]. This effect diminishes over time and normalizes to a certain degree after acclimatization. It seems as if both mechanisms combined impose the greatest impact on weight loss at altitude. Reduced appetite and enhanced energy expenditure lead to a negative energy balance and result in weight loss [12]. Both increased leptin expression and increased energy expenditure diminish with the degree of altitude acclimatization and supposedly come to a full stop after complete acclimatization. This initial period during altitude sojourns is characterized by a higher use of fatty acids recruited from the body’s reserves stored in adipocytes [10,47].

Chronic altitude exposure has been demonstrated to increase glucose uptake during exercise, while the consumption of leg free fatty acids was reduced during rest and exercise as well (these effects were potentiated by beta-blockade) [48]. More recently, acute hypoxia was shown to suppress exogenous glucose oxidation during steady-state aerobic exercise, but following altitude acclimatization, this suppression was reduced accompanied by sparing endogen carbohydrate stores and improving the negative energy balance [49].

Evidence for potentially elevated fatty acid oxidation in chronic hypoxia, defined as the phase of the hypoxic response where the protein expression of hypoxia inducible factors (HIF)-1α and HIF-2α decreased from the initial peak in acute hypoxia to a steady state level, comes from a study using monocytes [50]. These authors suggested that, in contrast to acute hypoxia, chronic hypoxia fuels the electron transport chain via electron-transferring flavoproteins, thereby elevating the production and consumption of fatty acids [50].

The decrease of fat tissue during altitude sojourns does not seem to be desirable considering its insulation effects and the resulting impairment in physical performance. Brown adipose tissue plays the most important role for insulation and thermoregulation in mostly cold high-altitude environments in animals and humans [51,52]. Cold, physical exercise (including shivering), leptin expression and interleukin-6 have a strong impact on adipocyte browning and thermogenesis. Especially in long-term altitude sojourns, this might be a factor which strongly increases energy expenditure. Those natural stressors initiate adipocyte browning towards beige fat, triggering a higher thermogenesis [53,54]. The role of hypoxia on the influence of the uncoupling protein 1 (UCP1), the most important mediator for thermogenesis in brown fat tissue, is controversial and seems to be dependent on the type of hypoxia. However, it seems from several studies in animals and humans that hypoxia influences UCP1 and thermogenesis in brown fat [55,56]. Notably, the role of possible transformation of white fat into brown fat tissue has not been taken into account in high altitude and hypoxia research enough so far. Recent research has discovered more and more gene expression pathways for the transformation of white fat tissue into brown fat tissue [57,58].

Lately, several different competing diets for a healthy lifestyle have been discussed [59,60,61,62,63]. The approach of comparing our current diet to evolutionary dietary forms has gained huge popularity and has been extensively discussed in popular magazines [64]. The supposedly oldest sample of dietary composition of a mountaineer is the one analyzed by Maixner et al. in the Iceman’s stomach. The composition of the Iceman’s last meal showed a very high portion of fat, manly brown fat from a hunted ibex [65]. The Iceman had his last meal a few hours before he died (most probably from an arrow shot at him), so he was already above 2500 m when he ate. It is unlikely that his last nutrition was by chance. The high-fat diet of the Iceman was likely based on his experience how to withstand cold and hypoxia at high altitude for the hike above 3200 m a.sl. Of course, he was well-acclimatized and prolonged exercise in cold (and hypoxic) environment may have contributed to this fuel selection. A high-fat diet has also been practiced by the native Inuit population supposedly providing a better protection against the cold [66]. In this context, more studies are warranted to investigate the effect of a high-fat diet on physical performance and weight loss during the first days of high-altitude sojourns. Although we do not have confirming studies for the success of such a diet approach, support comes from knowledge on adipocyte signaling in hypoxia.

In our opinion, it might also be advantageous to adapt to the diet several days or even weeks before ascent to prevent the additional challenge for the body to shift fuel utilization and metabolic processes during acclimatization.

Of course, we are aware of the limitations, especially related to the selected literature findings discussed and the lack of approaches at high altitude using high fat diets. However, our hypothesis is intended to stimulate such diet interventions in high-altitude sojourners. Furthermore, the resulting studies cover quite different research fields; nevertheless, the complexity of the phenomenon of weight loss during high-altitude sojourns requires a further look at the various influencing factors.

## 5. Conclusions

To our knowledge, this is the first structured review of the available literature regarding fat metabolism in multi-day high-altitude sojourns. The results are too inconsistent to provide a clear pathway of fat metabolism, including fat absorption or malabsorption, during multi-day high-altitude sojourns. The clearest statements might come from in vitro adipocyte studies and animal studies, that fat metabolism in hypoxia follows a certain time course depending on the level of HIF, which initiates further genes for leptin and other metabolic hormones. Whether the fat metabolism in humans at altitude exactly corresponds with these findings remains unclear. Individual fat and carbohydrate metabolism in single individuals, a topic becoming more and more popular in obesity research, as well as individual fitness and altitude levels, influence the results of high-altitude nutrition and metabolism studies. Therefore, a clear recommendation for nutrition for multi-day high-altitude sojourns cannot be given based on the results of this review. From the personal multi-day high- and extreme-altitude experiences of the senior authors on this paper, both forms of nutrition should be taken to avoid substantial energy loss: high carbohydrate supplements for the early days and high-fat sausages or ham/bacon for the later days of an expedition.

## Figures and Tables

**Table 1:** Brief summary of the main findings of the included articles.

Authors	Main Outcomes
Matu et al. (2017)	Changes in plasma acylated and total ghrelin concentrations may contribute to the suppression of appetite during a high-altitude trek
Devebec et al. (2014)	No change in appetite regulation but improved lipid profile after exercise training in normobaric hypoxia
McClelland et al. (1998)	Oxygen-sparing glucose oxidation at high altitude is outweighed by limited carbohydrate stores; exercise intensity is the primary determinant for fuel selection in rats
Horscroft et al. (2017)	Lower capacity of fatty acid oxidation is a major metabolic adaptation in Sherpas
Barbacini et al. (2019)	Serum sphingolipid concentration is the primary target of hypoxia adaptation in Andean children
Voss et al. (2014)	There are lower obesity rates when living at higher altitudes

## Data Availability

Data sharing not applicable. No new data were created or analyzed in this study.
